# Remarkable Enhanced Mechanical Properties of TiAlCrNbV Medium-Entropy Alloy with Zr Additions

**DOI:** 10.3390/ma15186324

**Published:** 2022-09-12

**Authors:** Po-Sung Chen, Sheng-Jia Shiu, Pei-Hua Tsai, Yu-Chin Liao, Jason Shian-Ching Jang, Hsin-Jay Wu, Shou-Yi Chang, Chih-Yen Chen, I-Yu Tsao

**Affiliations:** 1 Institute of Materials Science and Engineering, National Central University, Zhongli 320, Taiwan; 2 Department of Mechanical Engineering, National Central University, Zhongli 320, Taiwan; 3 Department of Materials Science and Engineering, National Yang Ming Chiao Tung University, Hsinchu 300, Taiwan; 4 Department of Materials Science and Engineering, National Tsing Hua University, Hsinchu 300, Taiwan; 5 Department of Materials and Optoelectronic Science, National Sun Yat-sen University, Kaohsiung 804, Taiwan

**Keywords:** medium-entropy alloy, lightweight material, solid solution strength, heat treatment, grain refinement

## Abstract

Most medium entropy alloys (MEAs) exhibit excellent mechanical properties, but their applications are limited because of their high density. This study explores a series of lightweight nonequiatomic Ti_65_(AlCrNbV)_35-x_Zr_x_ (x = 3, 5, 7, and 10) MEAs with a low density, high strength, and high ductility. To achieve solid solution strengthening, Zr with a large atomic radius was used. In addition, various thermomechanical treatment parameters were adopted to further improve the MEAs’ mechanical properties. The density of the MEAs was revealed to be approximately 5 g/cm^3^, indicating that they were lightweight. Through an X-ray diffraction analysis, the MEAs were revealed to have a single body-centered cubic structure not only in the as-cast state but also after thermomechanical treatment. In terms of mechanical properties, all the as-cast MEAs with Zr additions achieved excellent performance (>1000 MPa tensile yield strength and 20% tensile ductility). In addition, hot rolling effectively eliminated the defects of the MEAs; under a given yield strength, hot-rolled MEAs exhibited superior ductility relative to non-hot-rolled MEAs. Overall, the Ti_65_(AlCrNbV)_28_Zr_7_ MEAs exhibited an optimum combination of mechanical properties (yield strength > 1200 MPa, plastic strain > 15%) after undergoing hot rolling 50%, cold rolling 70%, and rapid annealing for 30 to 50 s (at a temperature of approximately 850 °C) with a heating rate of 15 K/s. With their extremely high specific yield strength (264 MPa·g/cm^3^) and high ductility (22%), the Ti_65_(AlCrNbV)_28_Zr_7_ MEAs demonstrate considerable potential for energy and transportation applications.

## 1. Introduction

Alloys have played a crucial role in the development of human civilization [1]. However, a traditional alloy comprises one major element as its matrix and other minor elements that are adjusted to improve its properties; this model considerably limits the development of alloy designs [2]. In 1996, high-entropy alloys (HEAs; also called multiprincipal element alloys [MPEAs]) were proposed as a novel alloy design. HEAs are characterized by multiple principal elements, and they exert unique effects that substantially overcome the limits of existing alloy designs [3,4,5]. Because their characteristics are different from those of traditional materials, HEAs exhibit superior properties relative to most conventional alloys. However, despite the excellent properties of HEAs, their density is usually >10 g/cm^3^ because of the high proportion of dense elements in these alloys [6,7]. Given that the application of HEAs is still considerably limited by various factors, the development of HEA designs that incorporate lightweight elements is necessary [8,9].

Because the designs of equiatomic HEAs still impose various restrictions in terms of alloy composition and properties, the concept of nonequiatomic HEAs have been proposed [10,11]. This innovative design not only retains the characteristics of HEAs but also substantially expands the scope of HEA designs [12]. Subsequently, nonequiatomic HEAs with multiphase structures and MEAs were proposed; these alloys exhibit favorable properties and retain the effects of HEAs, and they represent a new chapter in the design and development of future alloys [13,14,15]. In addition, nonequiatomic alloys are more flexible than equiatomic HEAs in terms of alloy composition adjustments, which are performed to prevent the formation of brittle intermetallic compounds [16,17]. Moreover, some studies showed that the MEAs present outstanding mechanical properties, which indicates that the MEAs possess a huge potential in the applications of industry [18,19,20].

In addition to alloy composition adjustments, the mechanical properties of nonequiatomic alloys can also be further improved through processes such as rolling or forging [21,22,23]. These alloys can be processed by deforming and annealing to modify their grain structure. The relationships among grain structure, strength, and ductility can also be regulated by controlling the heat treatment time to achieve high strength and ductility [24,25]. Additionally, the solid solution strengthening of these alloys can be achieved by the addition of minor atoms to enhance their strength; this is an effective method for improving their mechanical properties [26].

Studies of lightweight Ti-rich MEAs [27] have explored finetuned Ti_65_(AlCrNbV)_35_ alloy compositions and have added various amounts of atomic radius zirconium (Zr) to enhance the mechanical properties of these MEAs through substitutional solid solution strengthening. To further improve the mechanical properties of these MEAs, two thermomechanical processing routes have been tested; the first is cold rolling 70% (CR70) followed by rapid annealing, and the second is hot rolling 50% plus cold rolling 70% (HR50CR70) followed by rapid annealing. In addition, the relationship between the rapid annealing temperature and the microstructure and mechanical properties of MEAs was investigated.

## 2. Experimental Procedure

### 2.1. Materials

In the present study, high-purity raw materials (i.e., Al [99.99%], Ti [99.99%], Nb [99.99%], Cr [99.99%], V [99.9%], and Zr [99.99%]) were prepared to form Ti_65_(AlCrNbV)_35-x_Zr_x_ (x = 3, 5, 7, and 10) MEAs. Master alloys were fabricated by performing arc-melting in an argon atmosphere with a Ti-getter to prevent oxidation, and each master alloy was remelted four times to ensure alloy homogeneity. The master alloys were further made into ingots by performing tilt casting in an argon atmosphere. The dimension of each fabricated ingot was 40 mm (length) × 20 mm (width) × 10 mm (thickness). In addition, the ingots were homogenized at 1000 °C for 2 h in a high vacuum atmosphere (<10^−5^ torr) and then water quenched for subsequent thermomechanical treatment. To detect phase transformation within the working temperature range (25–1100 °C), a differential scanning calorimeter (DSC; STA 449 F3 Jupiter, Netzsch, Bavaria, Germany) was used at a heating rate of 10 K/min in an argon atmosphere.

### 2.2. Thermomechanical Treatment

Two types of rolling processes were performed; the first was cold rolling 70% (CR70), and the second was hot rolling 50% followed by cold rolling 70% (HR50CR70). During CR70, an ingot is rolled at room temperature with a deformation strain of 70%. During HR50CR70, an ingot is heated in the furnace at 1000 °C for 1 h and then rolled with a 50% strain. After a hot-rolled sample is cooled down to room temperature, it is then rolled at room temperature with a deformation strain of 70%. In addition, rapid annealing was performed in the present study. The effects of rapid annealing (heating rate of 15 °C/s) under various annealing durations (30, 40, 50, and 60 s) were examined to study the recrystallization sequence of MEAs and explore the relationship between their microstructure and mechanical properties.

### 2.3. Microstructure Characterization

The density of the MEAs was measured by applying Archimedes’ principle. X-ray diffraction (XRD; D2, Bruker, Billerica, MA, USA) was performed to identify crystal structures through Cu K_α_ radiation. Optical microscopy (OM; BX51M, Olympus, Tokyo, Japan) and electron backscatter diffraction (EBSD; HKL Channel 5, Oxford Instruments, Hobro, Denmark) were employed to characterize the microstructure of the MEAs. For XRD testing, the samples were ground and polished by silicon carbide sandpaper from #80 to #2000. For OM observation, the samples were polished with 0.3 and 0.05 µm Al_2_O_3_ polish suspension liquid after being ground and polished by sandpaper. For EBSD analysis, the samples were polished with an electro-polishing machine.

### 2.4. Mechanical Testing

A Vickers hardness tester (HV-100, Mitutoyo, Kawasaki, Japan) was used to measure the hardness of the studied MEAs under a load of 5 kg for 10 s. A universal testing machine (HT9102, Hung Ta, Taichung, Taiwan) was used to conduct tensile testing at room temperature under quasistatic loading with a strain rate of 1 × 10^−4^/s. The tensile testing samples were fabricated with a gauge dimension of 5 mm (length) × 2 mm (width) × 1.5 mm (thickness).

## 3. Results

The present study used the finetuned quinary lightweight Ti_65_(AlCrNbV)_35_ MEA that was developed from our previous study [27]. After the addition of a small amount of Zr (~160.3 pm), which has a large atomic radius that is different from that of the main element of Ti (~146.2 pm), the overall atomic size difference was calculated using Equation (1) [28], which is as follows:

(1) $$\delta =100\sqrt{\sum_{i=1}^{n}c_{i}{\left(1-r_{i}/\bar{r}\right)}^{2}}$$

where $\bar{r}=\overset{n}{\underset{i=1}{\sum }}c_{i}r_{i}$ and $c_{i}$ and $r_{i}$ represent the atomic fraction and atomic radius of the *i*th element, respectively. The total calculated atomic size differences are listed in Table 1. Notably, the atomic size difference *δ* increased with Zr additions, indicating that the effect of the solid solution increased when the Zr content was increased. Furthermore, the configuration entropy of the MEAs increased when the Zr additions were increased. Moreover, the atomic size differences (*δ*) of all the MEAs mostly approached 5%, which is conducive for the formation of a single solid solution [29]. Therefore, a series of Ti-rich MEA alloys such as Ti_65_(AlCrNbV)_32_Zr_3_ (Zr3), Ti_65_(AlCrNbV)_30_Zr_5_ (Zr5), Ti_65_(AlCrNbV)_28_Zr_7_ (Zr7), and Ti_65_(AlCrNbV)_25_Zr_10_ (Zr10) was explored.

### 3.1. Characterization of As-Cast Ti_65_(AlCrNbV)_35-x_Zr_x_ (x = 3, 5, 7, and 10) MEAs

The densities of the studied MEAs were measured by applying Archimedes’ principle. Table 2 reveals that the measured densities were similar to the theoretical densities on the basis of the mixing rule [27,28]. The measured density distribution was approximately 5.06–5.13 g/cm^3^, which met the requirement for a material to be lightweight (approximately 5 g/cm^3^).

**Figure 1:**
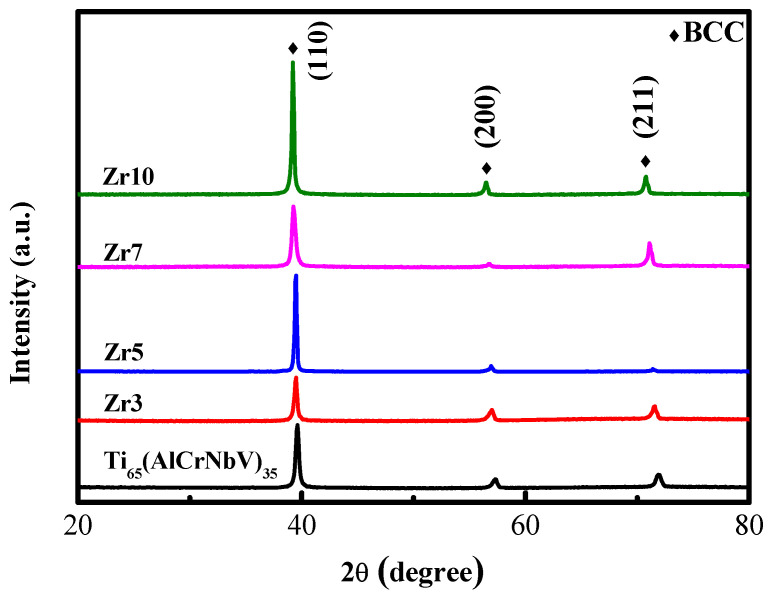
The XRD patterns of the as-cast Ti_65_(AlCrNbV)_35_ and Ti_65_(AlCrNbV)_35-x_Zr_x_ (x = 3, 5, 7, 10) MEAs.

**Figure 2:**
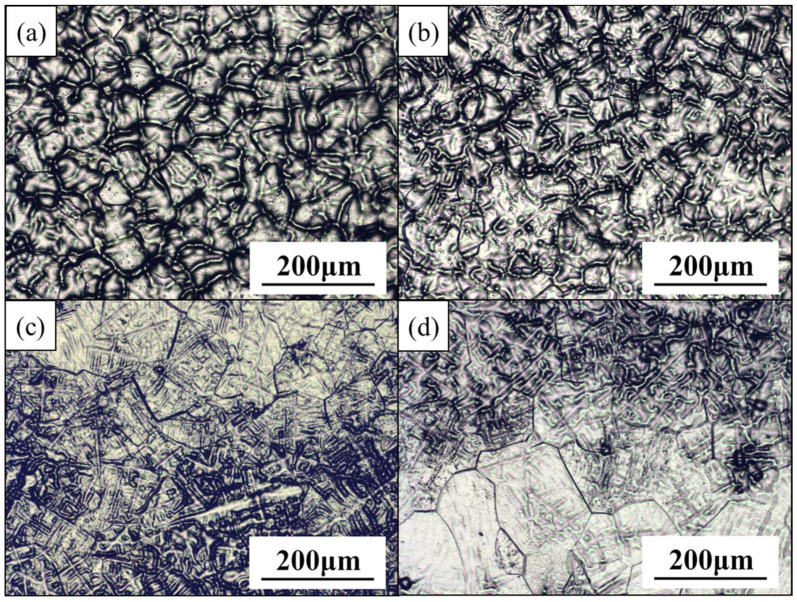
The appearance of the as-cast Ti_65_(AlCrNbV)_35-x_Zr_x_ (x = 3, 5, 7, 10) MEAs. (**a**) Zr3-100x; (**b**) Zr5-100x; (**c**) Zr7-100x; and (**d**) Zr10-100x.

Through an XRD analysis, the structures of the as-cast MEAs were revealed to contain a single body-centered cubic (BCC) phase (Figure 1). The diffraction peak shifted to the left when the Zr content was increased; this occurred because of the lattice distortion effect caused by the atomic size difference *δ* that resulted from the large atomic size of Zr, which was approximately 15–35 pm larger than the other elements in the alloy. This phenomenon was also reflected in the variation in the lattice constant, which increased when the Zr additions were increased (Table 1). In addition, the average grain size of the MEAs increased from 48 to 76 µm when the Zr content was increased (Figure 2), indicating that an increase in the Zr content tended to increase the grain sizes of the MEAs.

**Figure 3:**
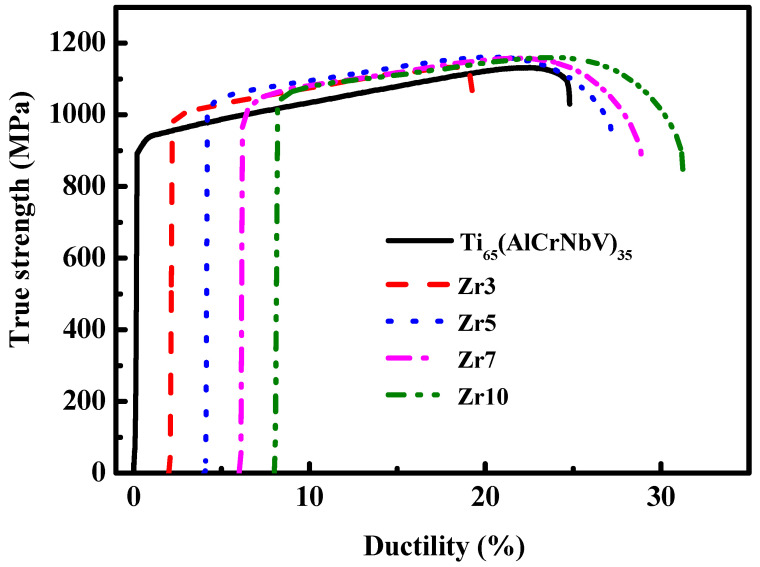
The tensile stress-strain curves of as-cast Ti_65_(AlCrNbV)_35_ and Ti_65_(AlCrNbV)_35-x_Zr_x_ (x = 3, 5, 7, 10) MEAs.

The identified mechanical properties of the as-cast MEAs are presented in Table 3 and Figure 3, which reveal that all the as-cast MEAs exhibited similar levels of hardness (approximately Hv 320–330). However, the yield strength and ductility of these as-cast MEAs exhibited an increasing trend when their Zr content was increased. The small increases in yield strength (approximately 3–8%) were consistent with the hardness variation results. The Zr additions were presumed to increase the grain sizes of MEA ingots, which reduced their yield strength and, therefore, compensated for the effect of solid solution strengthening achieved through Zr additions. Accordingly, to further improve the mechanical properties of the MEAs, their microstructure (e.g., fine grain or heterostructure) must be modified through thermomechanical treatment (TMT). Therefore, the as-cast Zr7 MEA, which had an optimum combination of yield strength and ductility, was selected for further processing through TMT.

### 3.2. Performance of the Zr7 MEA after TMT Processing

**Figure 4:**
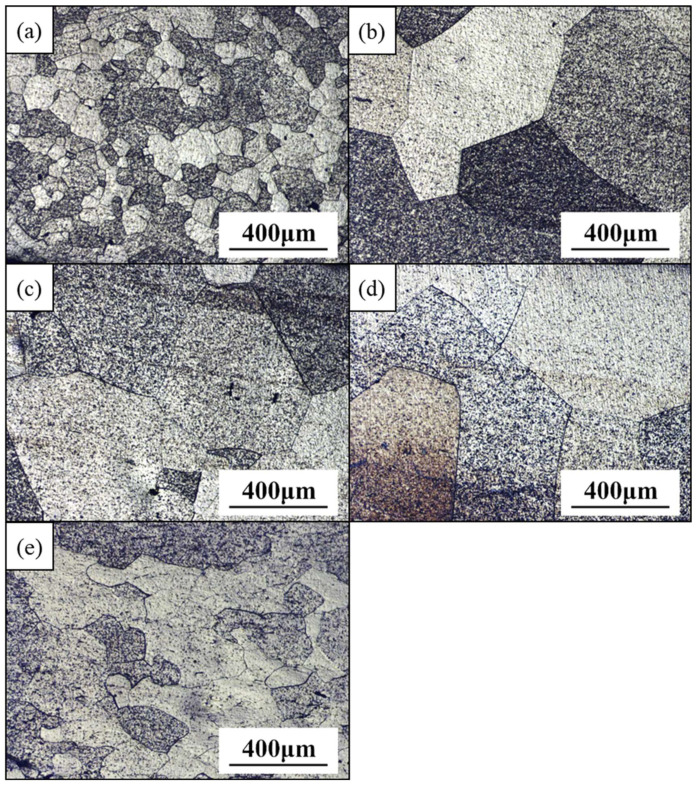
The appearance of the Ti_65_(AlCrNbV)_35-x_Zr_x_ (x = 3, 5, 7, 10) MEAs after homogenization heat treatment at 1000 °C for 2 h and hot rolling 50% at 1000 °C. (**a**) Zr3-homogenization; (**b**) Zr5-homogenization; (**c**) Zr7-homogenization; (**d**) Zr10-homogenization; and (**e**) Zr7-hot rolling.

Before they underwent TMT processing, the as-cast Zr7 MEA ingots were homogenized at 1000 °C for 2 h to eliminate composition inhomogeneity and dendritic microstructures. Figure 2; Figure 4 clearly reveal that the dendrites were effectively eliminated after homogenization and that the grain grew apparently from 83 to 485 μm.

**Figure 5:**
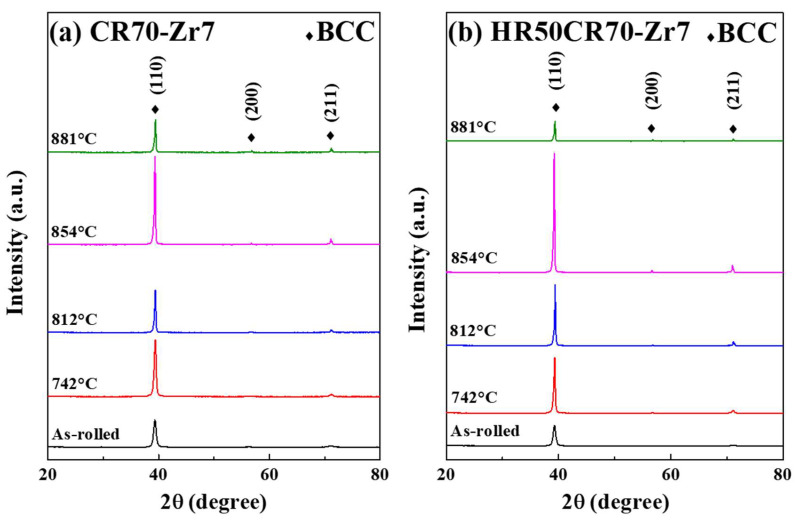
The XRD patterns of (**a**) CR70 and (**b**) HR50CR70 Ti_65_(AlCrNbV)_28_Zr_7_ MEAs with different annealing temperatures.

The homogenized Zr7 MEA ingots were further processed through TMT and rapid annealing; the samples underwent CR70 and HR50CR70 rolling processing, and they were then rapidly annealed for 30, 40, 50, and 60 s at a heating rate of 15 K/s to reach the temperatures of 742 °C, 812 °C, 854 °C, and 881 °C, respectively. Figure 4e suggests that the grain size of the Zr7 MEA after hot rolling was effectively reduced from 485 to 142 μm. The XRD results reveal that all the samples retained their single BCC structure irrespective of the applied processing route (Figure 5), indicating that the Zr7 MEA exhibits strong solid solution phase stability at high temperatures.

**Figure 6:**
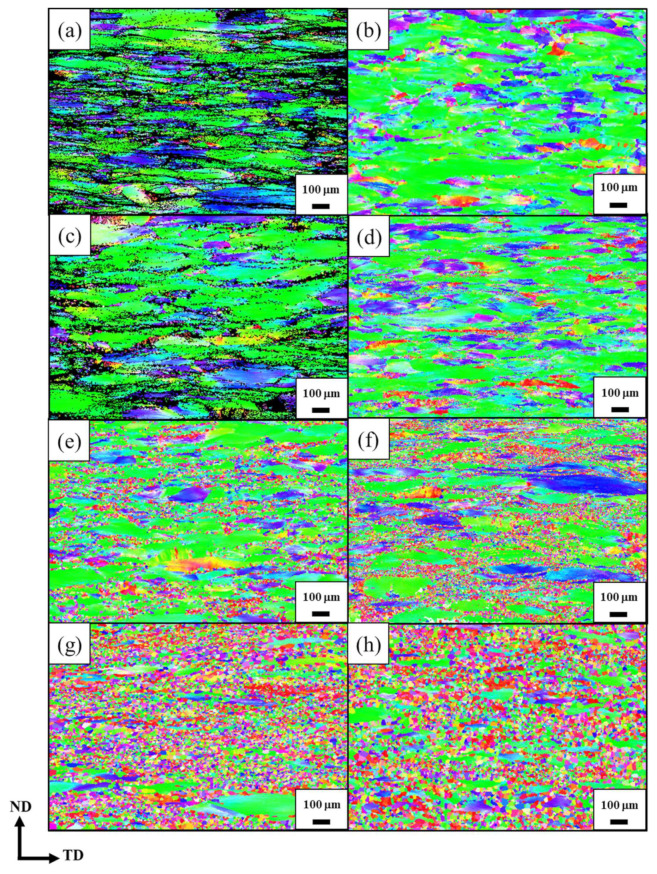
The EBSD images of CR70 and HR50CR70 Ti_65_(AlCrNbV)_28_Zr_7_ MEAs with different annealing temperatures. (**a**) CR70-742 °C; (**b**) HR50CR70-742 °C; (**c**) CR70-812 °C; (**d**) HR50CR70-812 °C; (**e**) CR70-854 °C; (**f**) HR50CR70-854 °C; (**g**) CR70-881 °C; (**h**) HR50CR70-881 °C.

Through an electron backscatter diffraction analysis, the recrystallization sequence of the Zr7 MEAs following TMT was observed clearly (Figure 6). A comparison of the processing routes of CR70 and HR50CR70 revealed that the larger plastic strain after cold rolling enabled this type of MEA to develop a stronger recrystallization ability. The Zr7 MEA samples started to recrystallize at 812 °C after undergoing HR50CR70 processing. By contrast, the initial recrystallization of the Zr7 MEA samples occurred at 854 °C after they underwent CR70 processing. Furthermore, at a given annealing temperature (e.g., 881 °C), the recrystallization area of the sample that underwent HR50CR70 processing was larger than that of the sample that underwent CR70 processing. A possible reason for this is that the grain size can be reduced effectively after hot rolling, and fine grains contain more boundaries that can accumulate more strain energy, which reduces the initial recrystallization temperature. Therefore, relative to the CR70 processing route, the HR50CR70 processing route provided more strain energy and nucleation sites and reduced the initial recrystallization temperature of the studied MEAs.

**Figure 7:**
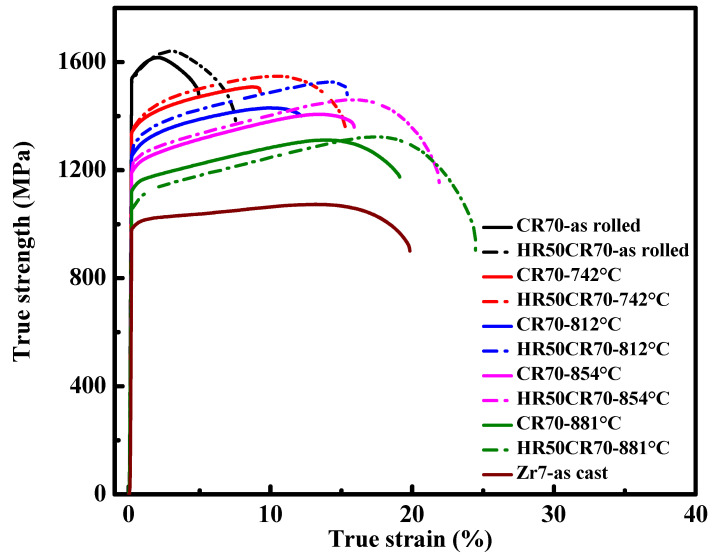
The mechanical tensile stress–strain curves of CR70 and HR50CR70 Ti_65_(AlCrNbV)_28_Zr_7_ MEAs with different annealing temperatures.

The results of tensile testing, which was conducted after the Zr7 MEAs underwent TMT processing, are presented in Figure 7 and Table 4. The yield strength of the as-rolled samples reached up to approximately 1530 MPa and was maintained under a plastic strain of approximately 5.0–7.5%. When the annealing time was increased while the heating rate was 15 K/s, the yield strength of the sample that was annealed for 30 s (sample temperature reached 742 °C) decreased considerably to approximately 1330 MPa. Subsequently, the slope of the decrease in yield strength started to flatten when the annealing time increased. The yield strength decreased to approximately 1250 MPa for the sample that was annealed for 40 s (the sample temperature reached 812 °C) and to approximately 1200 MPa for the sample that was annealed for 50 s (the sample temperature reached 854 °C). The small descending slope of the change in yield strength was attributed to the presence of recrystallized fine grains, which provided numerous grain boundaries that enhanced the yield strength and offset strain loss due to the recovery that occurred during the annealing process. Furthermore, the yield strength of the sample that was annealed for 60 s (sample temperature reached 881 °C) decreased to approximately 1100 MPa, indicating that the sample was almost fully recrystallized. By contrast, the ductility of this sample significantly increased by up to approximately 20%. 

On the basis of physical metallurgy, two factors can affect the recrystallization rate of HEAs; one is the rapid annealing temperature, and the other is the accumulation of strain energy. A higher annealing temperature allows for a sample to complete its recrystallization earlier, and it also coarsens recrystallized grains at a faster rate. A greater accumulation of strain energy also provides more driving force for enhancing the nucleation and grain growth rate of recrystallization. A comparison of the samples that underwent CR70 and HR50CR70 processing revealed that all the HR50CR70 samples exhibited greater ductility than the CR70 samples under as-rolled and annealed conditions. Hot rolling could have effectively eliminated defects after casting and refined grain sizes through dynamic recrystallization during hot rolling, resulting in the improved ductility of the studied MEAs. 

**Figure 8:**
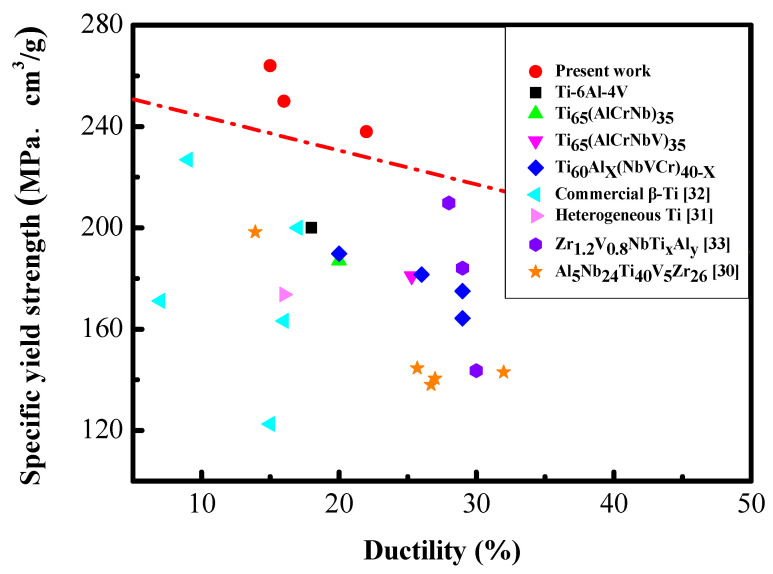
Comparison of specific yield strength and ductility [30,31,32,33]. Dashed line separates the data of the present work and previous reports.

Accordingly, among all the Zr7 MEA samples that underwent different processing routes, the samples that underwent HR50CR70 processing and were annealed for 30 to 50 s exhibited excellent combinations of yield strength (>1200 MPa) and ductility (>15%); notably, the Zr7 MEA that underwent HR50CR70 processing and was annealed for 30 s achieved a yield strength of 1350 MPa and a ductility of 15.3% (Figure 7 and Table 4). In Figure 8, the HR50CR70 Zr7 MEAs that are marked with red points exhibit a specific yield strength of 264 MPa·g/cm^3^ and a ductility of 15.3%, which are superior to those of commercial titanium alloys and other lightweight HEAs and MEAs [29,30,31,32].

## 4. Conclusions

A series of lightweight Ti-rich MEAs with minor additions of Zr were successfully fabricated through various TMT processing routes. On the basis of the experiment results, the microstructure evolution and mechanical properties of the alloys are summarized as follows:

The XRD results confirm that the structure of the Ti-rich MEAs maintained a single BCC phase not only when they were in an as-cast state but also after they underwent TMT processing.

The addition of Zr tended to increase the grain size of cast ingots and compensate for the effect of solid solution strengthening due to Zr additions. Therefore, although the increase in yield strength achieved by increasing the Zr of the as-cast alloys was nonsignificant, the ductility of the alloys remained mostly unchanged. 

The grain size can be refined effectively after hot rolling, and refined grains contain more grain boundaries that accumulate more strain energy, which reduces the initial recrystallization temperature. Therefore, the sample that underwent HR50CR70 processing had a low initial recrystallization temperature.

Under as-rolled and annealed conditions, the Zr7 MEA that underwent HR50CR70 processing exhibited greater ductility relative to the Zr7 MEA that underwent CR70 processing. Hot rolling could have effectively eliminated defects after casting and refined grain sizes through dynamic recrystallization, resulting in the improved ductility of the MEAs. 

The Zr7 MEA that underwent HR50CR70 processing and was annealed for 30 to 50 s at a heating rate of 15 K/s exhibited an excellent combination of yield strength (>1200 MPa) and ductility (>15%). In particular, the Zr7 MEA that underwent HR50CR70 processing and was annealed for 30 s achieved a yield strength of 1350 MPa and a ductility of 15.3%. Furthermore, the specific yield strength of Zr7 MEAs can reach up to 264 MPa·g/cm^3^. Compared to Ti-6Al-4V and other MEAs, the Zr7 MEA possesses a higher specific yield strength, which demonstrates the considerable potential for energy and transportation applications.

## Figures and Tables

**Table 1:** Parameters of as-cast Ti_65_(AlCrNb)_35_, Ti_65_(AlCrNbV)_35_, and Ti_65_(AlCrNbV)_35-x_Zr_x_ (x = 3, 5, 7, 10) MEAs [27].

Composition	ΔS (kJ. mol^−1^)	δ	Lattice Constant (Å)	Grain Size (µm)	
				As-Cast	Homogenization
Ti_65_(AlCrNb)_35_	8.58	4.69	3.226	81	124
Ti_65_(AlCrNbV)_35_	9.42	4.78	3.213	92	134
Ti_65_(AlCrNbV)_32_Zr_3_	9.92	5.04	3.227	48	100
Ti_65_(AlCrNbV)_30_Zr_5_	10.03	5.18	3.230	55	352
Ti_65_(AlCrNbV)_28_Zr_7_	10.07	5.29	3.243	70	416
Ti_65_(AlCrNbV)_25_Zr_10_	10.01	5.40	3.253	76	485

**Table 2:** The density of as-cast Ti_65_(AlCrNb)_35_, Ti_65_(AlCrNbV)_35_, and Ti_65_(AlCrNbV)_35-x_Zr_x_ (x = 3, 5, 7, 10) MEAs [27].

Composition	Theoretical Density (g/cm^3^)	Measured Density (g/cm^3^)
Ti_65_(AlCrNb)_35_	5.10	5.01 ± 1.79
Ti_65_(AlCrNbV)_35_	5.10	5.09 ± 0.19
Ti_65_(AlCrNbV)_32_Zr_3_	5.11	5.06 ± 0.98
Ti_65_(AlCrNbV)_30_Zr_5_	5.12	5.09 ± 0.58
Ti_65_(AlCrNbV)_28_Zr_7_	5.13	5.11 ± 0.39
Ti_65_(AlCrNbV)_25_Zr_10_	5.14	5.13 ± 0.19

**Table 3:** Tensile mechanical properties of as-cast Ti_65_(AlCrNb)_35_, Ti_65_(AlCrNbV)_35_, and Ti_65_(AlCrNbV)_35-x_Zr_x_ (x = 3, 5, 7, 10) MEAs [27].

Composition	Yield Strength (MPa)	Ultimate Strength (MPa)	Ductility (%)	Hardness (Hv)
Ti_65_(AlCrNb)_35_	954 ± 19	1136 ± 26	20.0 ± 1.6	323 ± 5
Ti_65_(AlCrNbV)_35_	921 ± 11	1159 ± 14	25.3 ± 1.4	317 ± 3
Ti_65_(AlCrNbV)_32_Zr_3_	976 ± 27	1134 ± 26	17.3 ± 2.6	337 ± 5
Ti_65_(AlCrNbV)_30_Zr_5_	1010 ± 13	1154 ± 25	20.1 ± 2.5	335 ± 4
Ti_65_(AlCrNbV)_28_Zr_7_	1015 ± 26	1170 ± 14	22.2 ± 2.2	331 ± 4
Ti_65_(AlCrNbV)_25_Zr_10_	1020 ± 9	1165 ± 22	23.9 ± 2.0	336 ± 3

**Table 4:** Tensile mechanical properties of Ti_65_(AlVCrNb)_28_Zr_7_ MEAs with different TMT parameters.

Processing	CR70			HR50CR70		
Mechanical Properties	Yield Strength (MPa)	Ultimate Strength (MPa)	Ductility (%)	Yield Strength (MPa)	Ultimate Strength (MPa)	Ductility (%)
As-rolled	1538 ± 23	1617 ± 19	5.0 ± 1.7	1528 ± 24	1640 ± 17	7.5 ± 1.7
742 °C	1334 ± 14	1508 ± 9	9.3 ± 2.1	1351 ± 11	1548 ± 5	15.3 ± 2.0
812 °C	1255 ± 8	1431 ± 11	12.2 ± 2.3	1279 ± 13	1527 ± 12	15.5 ± 1.3
854 °C	1192 ± 7	1407 ± 5	15.9 ± 2.7	1217 ± 17	1461 ± 8	21.9 ± 1.4
881 °C	1120 ± 14	1312 ± 6	19.2 ± 2.6	1052 ± 14	1324 ± 17	24.5 ± 2.2

## Data Availability

Data is contained within the article.
